# Perioperative Management of a Patient with Hemophilia C and Allergy to Fresh Frozen Plasma

**DOI:** 10.1155/2023/8973346

**Published:** 2023-03-29

**Authors:** Sara Kianian, Giacomo Scorsese, Eric Zabirowicz, Jeremy Poppers

**Affiliations:** Department of Anesthesiology, Stony Brook University Health Science Center, Stony Brook, NY 11794-8480, USA

## Abstract

Hemophilia C is a rare bleeding disorder characterized by a deficiency in clotting factor XI (fXI) and has no standard of care for preoperative optimization before cardiac surgery. Normalization of fXI levels in patients with hemophilia C can be achieved with fresh frozen plasma (FFP), which sometimes results in allergic reactions. We present a case of a patient with hemophilia C requiring coronary artery bypass grafting surgery who developed an allergic reaction to FFP. Our report underscores the balance between thrombosis and bleeding risks when devising a perioperative plan for patients with hemophilia C.

## 1. Introduction

Hemophilia C is a rare inherited bleeding disorder defined by a deficiency in clotting factor XI (fXI), that occurs in only about 1 : 1,000,000 people in the general population [1]. Affected individuals are generally asymptomatic, although significant bleeding may occur with trauma or surgery [2]. The utilization of systemic heparinization, sternotomy, extracorporeal circulation, and hypothermia during coronary artery bypass grafting (CABG) procedures results in significant trauma and disrupted hemostatic balance [3, 4]. Factor XI levels can be normalized prior to surgery with either fresh frozen plasma (FFP) or fXI concentrate. However, allergic reactions may occur with FFP administration and the use of fXI concentrate has been associated with thrombosis in patients with concomitant cardiovascular disease [5, 6]. Herein, we present a case of fXI deficiency in a patient who developed a rare allergic reaction to FFP prior to cardiac surgery. Before submission, consent for publication was obtained from the patient. This manuscript adheres to the applicable EQUATOR guidelines.

## 2. Case Description

A 75-year-old Caucasian male (height 170 cm and weight 82 kg) presented with exertional chest pain and an established history of coronary artery disease for which he had undergone a percutaneous intervention and drug eluting stent placement in the mid-left anterior descending (LAD) artery eight years prior. Other relevant medical history included severe fXI deficiency, otherwise known as hemophilia C, with deep muscle hematoma formation after minor trauma in the past, type 2 diabetes treated with oral antiglycemic medications, and hypertension. Cardiac catheterization revealed severe 3-vessel obstructive coronary artery disease for which revascularization with CABG was recommended.

**Figure 1:**
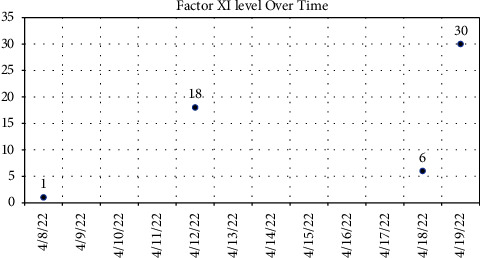
Factor XI activity levels as measured over time. Transfusions 1 and 2 with corresponding allergic reactions took place on 4/8/22. Transfusion 3 with pretransfusion medication to limit allergic reaction symptoms, a total of 2 units, took place on 4/11/22. Transfusion 4, similar to transfusion 3, but with 3 units in total took place on 4/12/22. The CABG took place on 4/19/22.

Preoperative laboratory values were significant for a platelet count of 77,000 (K/*μ*l), an activated partial thromboplastin time (aPTT) of 73.7 seconds (normal range 30–40 seconds), and a severely reduced fXI activity assay at <2% (normal >65%) (Figure 1). Mixing studies failed to demonstrate the presence of an inhibitor. Being that platelet dysfunction as well as heparin induced thrombocytopenia (HIT) are both common phenomena following cardiopulmonary bypass (CPB), the presence of thrombocytopenia prior was of important note. This value would be used to assess the need for platelet transfusions independent of managing his fXI deficiency.

In an attempt to raise the preoperative fXI activity level, the patient received four units of FFP followed by repeated coagulation studies and fXI activity assay. Soon after initiation of the first unit of FFP, however, the patient developed pruritic urticaria without evidence of angioedema or respiratory compromise. At this time, the transfusion was immediately stopped, and the patient was administered 50 milligrams of intravenous diphenhydramine, 20 milligrams of intravenous famotidine, and 650 milligrams of oral acetaminophen. The patient remained hemodynamically stable, without chest pain, and resolution of his allergic symptoms occurred shortly thereafter. At this point the hematology service was consulted and recommended that further transfusions be pursued at a slower rate of 15 cc per hour, along with pretreatment consisting of 50 milligrams of intravenous diphenhydramine, 20 milligrams of intravenous famotidine, and 650 milligrams of oral acetaminophen. Approximately 8 hours after the first transfusion, the second transfusion was initiated with the given recommendations. Within minutes, the patient again developed a pruritic rash with new onset expressive aphasia. The transfusion was aborted, the patient remained hemodynamically stable without evidence of respiratory compromise, and resolution of these symptoms occurred 30 minutes later.

**Figure 2:**
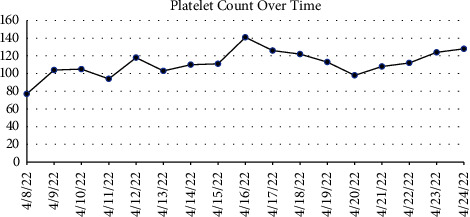
Platelet counts over time. Transfusions 1 and 2 with corresponding allergic reactions took place on 4/8/22. Transfusion 3 with pretransfusion medication to limit allergic reaction symptoms, a total of 2 units, took place on 4/11/22. Transfusion 4, similar to transfusion 3, but with 3 units in total took place on 4/12/22. The CABG took place on 4/19/22.

**Figure 3:**
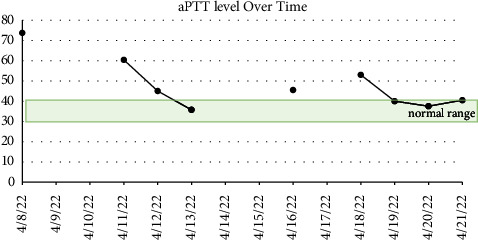
Activated partial thromboplastin time (aPTT) over time. Transfusions 1 and 2 with corresponding allergic reactions took place on 4/8/22. Transfusion 3 with pretransfusion medication to limit allergic reaction symptoms, a total of 2 units, took place on 4/11/22. Transfusion 4, similar to transfusion 3, but with 3 units in total took place on 4/12/22. The CABG took place on 4/19/22.

The following day, an interdisciplinary discussion ensued, which included representation from the allergy and immunology, hematology, cardiothoracic surgery, and cardiothoracic anesthesiology teams. The group considered the possibility of an immunoglobulin A (IgA) deficiency and need for IgA deficient donor FFP for subsequent transfusions, but laboratory investigation failed to identify an IgA deficiency. Moreover, the team considered alternative treatment modalities such as platelet transfusion alone and prothrombin complex concentrate (PCC). However, neither were pursued as PCC were deemed suboptimal for aPTT correction alone, and follow-up laboratory analysis revealed a correcting thrombocytopenia, without the need for platelet transfusion. Ultimately, the administration of 200 milligrams of intravenous hydrocortisone, 50 milligrams of intravenous diphenhydramine, 20 milligrams of intravenous famotidine, 650 milligrams of oral acetaminophen, and 10 milligrams of oral montelukast provided optimal pretransfusion therapy, allowing for the administration of 5 units of FFP over a period of ten hours without incident. Follow-up aPTT results were obtained with correction from presentation (70 to 36) and fXI activity increased to 25%, however it never approached normal values of greater than 65% (Figure 1). Ultimately, aPTT was considered more predictive of perioperative hemorrhage as the bleeding phenotype in hemophilia C is not well-correlated with fXI activity alone, but rather the presentation of bleeding events along with coagulation parameters such as aPTT [7]. Subsequently, the patient underwent a successful two-vessel CABG the following day (left internal mammary graft to the LAD and saphenous vein graft to the first obtuse marginal artery). Cross-clamp time was 63 minutes and total time on cardiopulmonary bypass time was 112 minutes. The estimated blood loss during the surgery was determined to be minimal by the surgical team, with a total of 2.6 L of washed fluids yielding 264 milliliters of cell-saver-packed red blood—which was returned to the patient at the conclusion of surgery. Hemoglobin concentrations preoperatively, intraoperatively, and postoperatively were also tracked (Figures 2 and 3). The patient was extubated 3 hours after arrival in the cardiothoracic intensive care unit and subsequently discharged four days thereafter.

## 3. Discussion

Factor XI is one of many clotting factors required for the propagation of thrombin generation and is one of the critical factors needed to induce the thrombin burst for coagulation [8]. While a necessary factor for clinical assays, such as aPTT and fXI has been described to have a variable role in clinically significant bleeding for patients with fXI deficiency, otherwise known as hemophilia C [9]. Hemophilia C or fXI deficiency is a rare bleeding disorder with an incidence suspected to be only 1 : 1,000,000 in the general population [1]. Although rare, this condition requires careful monitoring and management, especially for patients undergoing surgery or otherwise has some provocation of bleeding. Given that the circulating half-life of fXI is known to be between 60 and 80 hours, it is relevant to reassess coagulation parameters at least every two to three days prior to major procedures with a risk of bleeding [10]. As such, optimizing coagulation parameters is imperative in the perioperative period to limit excessive bleeding and complications.

Treatment of patients with hemophilia C undergoing surgery has been achieved with the use of FFP until target laboratory parameters are achieved, both preoperatively and with intraoperative assessments [3, 11]. Other reported treatment modalities for cardiac surgery cases, include purified fXI replacement, activated recombinant factor VIIa, desamino-8-D-arginine vasopressin, and antifibrinolytic agents such as tranexamic acid [6, 12–19]. The alternative treatment modalities utilizing recombinant factor VIIa or fXI replacement therapy, when used in patients with existing cardiac disease, carries a prothrombotic risk and must be carefully chosen for treatment based on the clinical picture, such as in patients who have an fXI inhibitor and thus cannot receive FFP transfusions [7, 20]. Furthermore, excessive FFP transfusions carry the risk of circulatory overload [5]. Unique to this report is that our patient developed a rare atopic reaction to FFP, with no known inhibitor of IgA deficiency present. With this negative workup and an unknown cause for the hypersensitivity response to the FFP, further reactions could be present to other plasma products such as platelets, cryoprecipitate, and packed red blood cells. Therefore, in preparation for potentially significant bleeding, a delicate balance was struck between mitigating perioperative coagulopathy with the concomitant concerns for an allergic transfusion reaction. Ultimately, we chose to optimize the aPTT levels using a combination of pretreatment with diphenhydramine, acetaminophen and hydrocortisone, as well as slow administration of FFP over the course of hours. Through this technique, our team was able to successfully address our patient's coagulopathy through the normalization of aPTT, which has been demonstrated to correlate more closely to the risk of clinically significant bleeding than fXI levels [3, 7].

Hemophilia C is a rare bleeding disorder that lacks clear guidelines for management of patients undergoing surgery. A critical component to our perioperative management was the careful use of premedication utilizing diphenhydramine, acetaminophen, and hydrocortisone, as well as slow administration of FFP. This would ultimately allow our team to strike a balance between achieving preoperative hemostasis and mitigating potential adverse events related to this patient's rare allergic reaction to FFP. Ultimately, extensive collaborative efforts between the cardiothoracic surgery, cardiothoracic anesthesiology, hematology, and allergy and immunology teams resulted in a clear preoperative plan allowing for the normalization of the aPTT and, finally, for the CABG to be performed without complication or excessive bleeding.

## Data Availability

The data used to support the findings of this study are included within the article.

## Abbreviations

fXI: Factor XI
FFP: Fresh frozen plasma
CABG: Coronary artery bypass grafting
LAD: Left anterior descending
aPTT: Activated partial thromboplastin time
IgA: Immunoglobulin A
PCC: Prothrombin complex concentrate.
